# Collaborating sites for community-oriented integrated care and health promotion

**DOI:** 10.1080/17571472.2016.1271491

**Published:** 2017-01-09

**Authors:** Paul Thomas

**Affiliations:** Editor LJPC

**Keywords:** Community-oriented integrated care and health promotion, new care models

## Abstract

London Journal of Primary Care wishes to develop a network of collaborating sites to better understand how to achieve *community*-*oriented integrated care and health promotion* in different contexts. A collaborating site can do more than submit papers. It can develop its own domain on the LJPC website, contribute to the development of LJPC policy, and stimulate discussions with other collaborating sites. At any time a collaborating site can opt out. In addition to securing papers for publication, a site might nurture a network of supporters, teach people to use multiple research and quality improvement methods, develop a system of governance for locally led inquiries, develop case studies of community-oriented integrated care and health promotion and facilitate within-site and between-site learning and change.

## Why this matters to me

*Community*-*oriented integrated care and health promotion* is a way to achieve the Five Year Forward View. It bears similarity to community-oriented primary care, and *strong primary health care* as envisaged at the 1978 WHO Alma Ata Declaration. I want to make it easier for primary care practitioners to explore what it might practically mean in different contexts.

## Key message

A LJPC collaborating site nurtures papers for publication and builds local capacity to inquire and change.

London Journal of Primary Care (LJPC) wishes to develop a network of collaborating sites to better understand how to achieve *community*-*oriented integrated care and health promotion* (COIC) in different contexts. Participants at the 2015 London City Health Conference led by the RCGP London Faculties considered COIC to be an important goal [1].

## What is COIC?

COIC is inspired by the 1978 WHO Alma Ata Agreement that all citizens need to contribute to healthy communities [2] and the Five Year Forward View that calls for *new partnerships with local communities* [3]. COIC is about much more than general practice as we know it. It includes partnerships between primary care, public health and many others to advocate for whole society collaboration for healthy environments as well as shared care and self-care for those who are unwell.

Community-oriented integrated care and health promotion (COIC) is shared care and collaborative health promotion at local, community level. The word ‘local’ is important, because it’s easier to see the range of factors that affect people’s health when they are in everyday, real-life situations. ‘Community’ is important because it’s easier to collaborate for healthy society when people feel bound together with trusted relationships. If we step away from a local or community level, our focus narrows. For example, in a hospital the focus of attention is the reason for being there – usually treating a disease.

The addition of the term ‘health promotion’ is important because without it we may be tempted to think of integrated care solely in terms of care for people with medical conditions. You have to think differently when considering someone’s health and when considering their diseases. Both are needed. We need people to be able to distinguish between them and use both routinely.

COIC prevents the inefficiencies of fragmented care. It builds boundary-spanning teams, networks and communities for both health and care [4]. It builds local health communities and networks [5]. It shows how individuals, organisations and networks can integrate their work with that of others by obeying simple rules: *Relate to the same geographic areas and the same seasons of activity. Facilitate cycles of collaborative learning and coordinated change to enable organic co*-*evolution. Set discrete activities like treating diseases within more complex things like improving people’s health.* These rules help people to connect more like organic cells in a body than mechanical links in a chain.

Since 1978, healthcare integration throughout the world has emphasised care pathways for diseases. The value of broader integration of efforts for a healthy society has always been recognised, but it has never been realised at scale [6]. One reason for this is that every place is different and the principles of COIC will find different expression in different places. Hence the importance of case studies that show how to apply the principles of COIC in different ways in different contexts (see partner paper on case studies) [7].

COIC does many things at the same time. It makes it easier to treat diseases, through teamworking between community-based practitioners, with timely specialist input. Shared care, care plans, self-care and shared records all help to do this. Sometimes people with illnesses need to go to specialists, but specialist expertise can also be brought into local consultations through telephone, video and email. This kind of teamworking is sometimes called ‘vertical integration’ and is evident in the 2016 NHS ‘New Care Models’ that integrate Primary and Acute Care Systems (PACS) [8].

As well as treating diseases, COIC improves health of both individuals and whole communities. Being healthy means being able to build mutually supportive relationships that lead to healthy individuals, healthy families and healthy communities [9]. This kind of teamworking for a healthy society is sometimes called ‘horizontal integration’ and is evident in the 2016 NHS ‘New Care Models’ of Multi-speciality Community Provider (MCP) that integrate care through locally based ‘care hubs’ [8].

COIC resembles community-oriented primary care [10,11]. They both build communities around general practices. They both require partnership–working with public health and others. The difference is that COIC revolves around geographic areas rather than individual general practices. This allows other organisations to lead parallel initiatives for health and care in the same area to build a local community for health and a culture of collaboration. General practice has, in partnership with public health, a crucial role in encouraging these.

In COIC, leadership teams span organisational boundaries [4] to lead annual cycles of collaborative inquiry and coordinated improvement. Such participation produces a sense of belonging and provides opportunities for people of very different backgrounds to get to know each other, stimulating innovation, friendships and teamworking throughout whole systems of care. *Participatory action research* [12] and *participatory research* [13] are established methodologies that do this. Broad participation helps to develop complex interventions [14] make sense of multi-methods approaches to quality improvement [15].

Meads’ 31 country study reveals *ideal types* of organisation [16] that help to understand combined horizontal and vertical integration in COIC [17,18]. Such combination is evidenced, for example, in models of diabetes care that link general practice with specialists in the vertical direction and with extended primary care teams in the horizontal direction [19]. Similarly, good strategy for mental health combines (vertical) treatment for ill health and (horizontal) mental health promotion [20].

COIC requires non-medical perspectives [21] and community development approaches to primary care development [22]. It helps us to rediscover the founding principles of the NHS, that good care for everyone is preferable to outstanding care for a few [23], and to consider ethical and spiritual aspects of health [24]. It provides a way to apply in a modern context the principles of quality general practice worked out by visionaries of yesterday [25,26].

To shape locally relevant models of COIC we need to think about organisations and systems and not merely about individuals [27–29]. We need to explore ways that *learning organisations* [30] can address the weaknesses of the small business model of general practice and the bureaucratic model of large organisations. And we need to learn from other countries [4,5,13,16,23,31–38], and take a global perspective [39]. We need to consider ‘up-stream’ learning needs – school and university students of all ages need to know how to develop relationships, communities and systems, and see the limitations of models of individual excellence.

## What should LJPC Collaborating Sites do?

A collaborating site should support the publication of papers from their areas to help understand different aspects of COIC in different contexts. They need to be mindful that specific models are bound by the local context, whereas principles are more generalisable. So papers need to describe the local context and the rationale for the models that they evaluate. These papers do not need to be published in the LJPC. They can be published in other journals or books, or used solely for local learning. What the LJPC can offer is a network of people who want to share learning about such work; LJPC can also publish summaries of papers about those sites published in other places.

Anyone can submit papers to LJPC, not merely collaborating sites. Those that pass the peer review process are published either as ‘Reviewed Papers’ (submitted to PubMed for academic citation) or as ‘Landscape Papers’ (not submitted to PubMed but searchable by search engines like Google). A collaborating site can do more than submit papers. It can develop its own domain on the LJPC website, contribute to the development of LJPC policy, and stimulate discussions with other collaborating sites. At any time a collaborating site can opt out. There is no payment. You get involved because it works for you.

Collaborating sites will need some kind of ‘Unit’ to provide linkage with other sites. The Unit might be an existing group or a newly convened one. It can be called whatever makes sense locally – a Steering Group, a Satellite LJPC Board, or perhaps most logically an *Applied Research Unit*, because it will aim to publish local work in ways that also increase local capacity to evaluate complex service improvements. The Unit is likely to include academics and practitioners of different disciplines, policy-makers and others who want to improve the integration of healthcare in their area. It is probably best to be hosted by an organisation that has the authority to engage a broad group of stakeholders, for example a university department, clinical commissioning group or health centre.

These Units might shape local strategy for research, audit and evaluation. Through LJPC and others the network of sites can stimulate broader discussions about an authentic primary care research agenda that has the power to illuminate the complex and ever-changing nature of the ‘real world’. The contributions can be small, perhaps submitting occasional papers for publication, or large, becoming a semi-independent journal under the umbrella of LJPC. You can start small and see how things go.

## What other things might LJPC Collaborating Sites aspire to do?

In addition to securing papers for publication, a Unit might consider the following activities:

(1) *Nurture a network of members.* Send a regular, e.g. monthly update to a network of stakeholders to build a research community around the collaborating site. Use local resources to develop leaders, action researchers and authors.
(2) *Teach people to use multiple research and quality improvement methods.* The inquiry paradigms of positivism, critical theory and constructivism reveal different aspects of complex dynamic situations [39]. A combination of methods from these paradigms can reveal whole stories as well as discrete facts [40]. *Participatory action research* [12] and *case studies* [7] are useful methodologies to combine research insights and local experience.
(3) *Develop a system of governance for locally* *led inquiries.* A local facility to support multidisciplinary leadership of whole system improvements can help researchers to ask good research questions and use rigorous methods. Participants at learning sets can learn about research methods and about leadership skills. This can result in a network of change-agents that can build capacity to research and write papers about primary care innovation. Leadership courses can link with policy-making processes to enable annual cycles of collaborative learning and coordinated improvement.
(4) *Develop case studies of community-oriented integrated care and health promotion.* By amalgamating data to the same geographic areas [41,42] and facilitating locality-based stakeholder workshops to review improvements it is possible to build case studies of community-oriented integrated care and health promotion. Networks of case studies can learn from and with each other to facilitate change across much larger areas.
(5) *Facilitate within-site and between-site learning and change.* Local workshops can bring together research, audit and evaluated service improvements to support co-design of service developments. Large group events like Real-Time Strategic Change and Open Space can support cultural change towards integrated working [43]. In addition to LJPC networks, learning can be shared between sites through established learning networks, including NHS England’s clinical networks, Healthy London Partnerships, Academic Health Science Networks, and National Institute for Health Research Collaboration for Leadership in Applied Health Research and Care West (CLAHRCs).

Collaborating sites could become a force for local innovation and capacity improvement as well as nurturing papers for publication. They could help to develop individual leaders and multidisciplinary leadership teams that include practitioners of various disciplines, researchers, managers and policy-makers. They could help to develop a primary care research agenda, and a language for integrated working and collaborative health promotion, that translates to the modern world the vision of Alma Ata and the traditional values of whole person, family and community-oriented general practice.

## Conflict of interest

Paul Thomas has written a book about community-oriented integrated care and health promotion, to be published in 2017.
